# Malaria over-diagnosis in Cameroon: diagnostic accuracy of Fluorescence and Staining Technologies (FAST) Malaria Stain and LED microscopy versus Giemsa and bright field microscopy validated by polymerase chain reaction

**DOI:** 10.1186/s40249-017-0251-0

**Published:** 2017-04-04

**Authors:** Sean M. Parsel, Steven A. Gustafson, Edward Friedlander, Alexander A. Shnyra, Aderosoye J. Adegbulu, Ying Liu, Nicole M. Parrish, Syed A. Jamal, Eve Lofthus, Leo Ayuk, Charles Awasom, Carolyn J. Henry, Carole P. McArthur

**Affiliations:** 1 Department of Pathology, Kansas City University of Medicine and Biosciences, 1750 Independence Ave, Kansas, MO 64106 USA; 2 Department of Pharmacology and Microbiology, Kansas City University of Medicine and Biosciences, 1750 Independence Ave, Kansas, MO 64106 USA; 3 Royal Veterinary College, 4 Royal College St, London, NW1 0TU UK; 4 Department of Biostatistics and Epidemiology, East Tennessee State University, P.O. Box 70259, Johnson, TN 37614 USA; 5 Department of Pathology, Division of Microbiology, Johns Hopkins Medical Institute, Meyer B1-193, 600 North Wolfe Street, Baltimore, MD 21287 USA; 6 Rockhurst University, 1100 Rockhurst Rd, Kansas, MO 64110 USA; 7 University of Missouri-Kansas City, School of Dentistry, 650 E 25th Street, Kansas, MO 64108 USA; 8 Cameroon Ministry of Health Regional Hospital, Bamenda, Cameroon; 9 Department of Veterinary Medicine and Surgery, University of Missouri, 900 East Campus Drive, Columbia, MO 65211 USA; 10 Department of Otolaryngology, Tulane University School of Medicine, 1430 Tulane Ave, New Orleans, LA 70115 USA

**Keywords:** Malaria, Fluorescent microscopy, Giemsa, Diagnostic accuracy

## Abstract

**Background:**

Malaria is a major world health issue and its continued burden is due, in part, to difficulties in the diagnosis of the illness. The World Health Organization recommends confirmatory testing using microscopy-based techniques or rapid diagnostic tests (RDT) for all cases of suspected malaria. In regions where *Plasmodium* species are indigenous, there are multiple etiologies of fever leading to misdiagnoses, especially in populations where HIV is prevalent and children. To determine the frequency of malaria infection in febrile patients over an 8-month period at the Regional Hospital in Bamenda, Cameroon, we evaluated the clinical efficacy of the Flourescence and Staining Technology (FAST) Malaria stain and ParaLens Advance^TM^ microscopy system (FM) and compared it with conventional bright field microscopy and Giemsa stain (GS).

**Methods:**

Peripheral blood samples from 522 patients with a clinical diagnosis of “suspected malaria” were evaluated using GS and FM methods. A nested PCR assay was the gold standard to compare the two methods. PCR positivity, sensitivity, specificity, positive predictive value (PPV), and negative predictive value (NPV) were determined.

**Results:**

Four hundred ninety nine samples were included in the final analysis. Of these, 30 were positive via PCR (6.01%) with a mean PPV of 19.62% and 27.99% for GS and FM, respectively. The mean NPV was 95.01% and 95.28% for GS and FM, respectively. Sensitivity was 26.67% in both groups and specificity was 92.78% and 96.21% for GS and FM, respectively. An increased level of diagnostic discrepancy was observed between technicians based upon skill level using GS, which was not seen with FM.

**Conclusions:**

The frequency of malarial infections confirmed via PCR among patients presenting with fever and other symptoms of malaria was dramatically lower than that anticipated based upon physicians’ clinical suspicions. A correlation between technician skill and accuracy of malaria diagnosis using GS was observed that was less pronounced using FM. Additionally, FM increased the specificity and improved the PPV, suggesting this relatively low cost approach could be useful in resource-limited environments. Anecdotally, physicians were reluctant to not treat all patients symptomatically before results were known and in spite of a negative microscopic diagnosis, highlighting the need for further physician education to avoid this practice of overtreatment. A larger study in an area with a known high prevalence is being planned to compare the two microscopy methods against available RDTs.

**Electronic supplementary material:**

The online version of this article (doi:10.1186/s40249-017-0251-0) contains supplementary material, which is available to authorized users.

## Multilingual abstracts

Please see Additional file 1 for translations of the abstract into the five official working languages of the United Nations.

## Background

Malaria is an ongoing threat to world health, leading to over 600 000 deaths each year despite increasing awareness and public health efforts directed at controlling the disease. In Cameroon, malaria is perennial, rainfall-dependent and the prevalence is 42.5% in children under 5 years of age, 31.5% in those between 5 and 15 years of age, and 10.5% in those older than 15 years. It causes 50% morbidity in children under 5 years old, and is implicated in 40% to 50% of medical consultations [1]. The predominant species in the region is *Plasmodium falciparum* [2]. Early diagnosis with reliable microscopic confirmation and effective treatment is paramount in reducing morbidity and mortality [3]. While malaria is a preventable disease, there are seemingly insurmountable issues with proper diagnosis and treatment throughout endemic regions, leading to its continued prevalence and increasing drug resistance. Due to inadequate resources, inexperienced technical personnel, poor diagnostic standards, and the lack of clinician confidence in diagnostics currently available, the disease may be misdiagnosed and individuals treated unnecessarily with antimalarial agents.

The current World Health Organization (WHO) recommendation is detection of parasites using bright field microscopy or rapid diagnostic tests (RDTs) before making a diagnosis and implementing treatment [4]. Despite this, in regions where malaria is endemic, less than half of the individuals with presumptive malaria will receive confirmatory testing and will, instead, receive empiric therapy [3]. Through policy changes and implementation of RDTs, diagnostic testing has improved, although testing in febrile children is still lacking [5]. Limited access to appropriate resources, as well as social factors, contribute to the difficulty of diagnosis in patients presenting with symptoms of malaria. This is especially true for children. Some individuals in resource-limited regions with febrile illness pursue informal private sector care at pharmacies, traditional healers, or marketplace therapies instead of formal medical care. Those who seek care at local clinics are often treated empirically due to lack of reliable tests and financial concerns that prohibit transport to government run facilities where these resources may be present [5, 6]. Often, in remote areas where a microscope may not even be available, diagnosis is empirical and based solely on clinical features. This approach may lead to overtreatment of malaria, missed diagnosis or inappropriate treatment of other febrile illnesses, wasting of resources, and development of drug resistance secondary to selective pressure against the *Plasmodium* parasite [4, 7–12]. The reliance on clinical features to diagnose malaria is consistent with the widespread practice of treating all febrile illness as malaria based on the presumptive diagnosis in endemic regions with high prevalence. However, especially in the era of HIV and the associated opportunistic infections, the signs and symptoms of malaria may be vague and the clinical presentation may be limited to a history of fever [13]. Due to the nonspecific clinical picture, many other causes of fever should be considered in addition to malaria [4]. In 2014, D'Acremont et al. showed that the majority of cases of fever of unknown origin in regions where malaria is endemic are caused by viral or bacterial illnesses and not malaria [14]. Despite this, a large majority of individuals continue to receive unnecessary treatment with antimalarial medications, owing to the difficulty in changing long-standing clinical habits and practices. With the possible exception of infants at risk for cerebral malaria, it is vital to confirm a diagnosis of malaria prior to treatment. This approach can circumvent futile treatment and reduce the development of drug resistance, An appropriate diagnostic test is one that is cost-effective, accurate, and can produce results within a time frame appropriate to the severity of the clinical circumstances [4, 15].

Currently, the gold standard for malaria diagnosis is the use of microscopy-based methods such as bright field microscopy with Giemsa-stained specimens (GS) or by validated RDT when available. However, the lack of clear policies within some institutions, varied behaviour of individual clinicians, lack of quality control, and inadequate resources may result in poor outcomes [3]. In order to ensure accuracy, laboratory technicians must be properly trained and there must be effective quality control and quality assurance programs in place; however this is often not possible in our experience [16]. A recent survey suggests that many laboratories in Sub-Saharan Africa lack quality control and many are not WHO-accredited [17]. The poor quality of some clinical laboratories leads to a negative impact on healthcare as a whole, perpetuating misdiagnoses and decreasing clinician and community confidence in the use of such services. The questionable reliability of microscopic techniques in the region leads to decreased utilization of laboratory-based methods. As a result, millions of individuals with suspected malaria do not receive the recommended tests prior to therapy [3]. The current study was prompted by observations that most patients presenting with fever were automatically receiving treatment for malaria. Thus, there was an urgent need to determine the number of malaria-positive fevers and evaluate the status of the current testing at the Bamenda Regional Hospital, which is located in the high grasslands of the northwest province of Cameroon.

The use of fluorescent-based microscopy techniques can be superior to GS in terms of sensitivity and have been successful in detection of *Mycobacterium tuberculosis* [18]. However, conventional fluorescence microscopy is expensive and requires a costly microscope and technical skill, making this method impractical in resource-scare regions [19–21]. In light of this and from our experience, we elected to evaluate inexpensive LED fluorescent attachments for light microscopes that allow the conversion of a standard microscope into an epifluorescence microscope at a fraction of the cost of conventional methods. We chose the ParaLens Advance^TM^ light microscope attachment (QBC Diagnostics, Port Matilda, PA) since it was a system we recently evaluated against a standard light microscope for diagnosis of *Mycobacterium* infections and observed 100% agreement between both methods [18]. The use of this relatively inexpensive diagnostic system and staining method has not been applied in cases of malaria to our knowledge. The majority of published studies using this technique have been directed toward detection of *Mycobacterium* species and have shown positive results and increased sensitivity in comparison to standard methods [22, 23]. A similar method is available for the detection of malaria infections using parasite-specific stains such as the Fluorescence and Staining Technologies (FAST) Malaria stain produced by the company QBC Diagnostics. This is not to be confused with an alternative technique in vogue during the 1980s referred to as the QBC (quantitative buffy coat) test that requires a specific centrifuge to separate *Plasmodium* organisms in capillary tubes and is, therefore, more expensive. The main advantages of the FAST Malaria stain over GS are that it is presumably less technically complex, faster to perform, can be used with a relatively inexpensive LED microscopy attachment, obviates the need for a halogen lamp or disposing of the heavy metals, and may require less training and experience to make an accurate diagnosis. Because of these advantages, such a technique could be employed in developing countries where malaria is endemic in order to improve diagnostic accuracy without a large increase in costs.

In the current study, we evaluated the performance of the FAST Malaria stain and ParaLens Advance^TM^ microscope system (FM) in comparison to GS in a prospective study in Cameroon. Polymerase chain reaction (PCR) was used to confirm the presence of blood-borne *Plasmodium* species and as the standard for comparison between the two microscopy methods. The objectives of this study were to determine the frequency of malaria in patients presenting with fever and to compare the sensitivity, specificity, negative predictive value (NPV), and positive predictive value (PPV) of the two basic staining methods.

## Methods

This study was conducted in accordance with the principles outlined in the Helsinki Declaration and ethical approval was received in February 2011 from the University of Missouri-Kansas City Institutional Review Board (IRB) and the Cameroon Ministry of Health Regional Hospital IRB in Bamenda, Cameroon. Training of the technicians in the FM technique and reagent quality control was executed the week prior to the initiation of the study until there was less than 10% difference in skill between results using known semi-quantitative 1+, 2+, and 3++ malaria control slides. The collection and staining of slides and samples for PCR were in parallel to routine standard of care (Giemsa) at the Regional Hospital over an 8-month period between January and August 2011. Samples spotted in quadruplicate onto filter paper cards for PCR analysis were transported by air to the United States in 2012 and analysed in a blinded fashion at Kansas City University of Medicine and Bioscience between 2013 and 2014.

### Selection criteria and specimen collection

Peripheral blood samples were obtained sequentially from voluntary participants with a clinical diagnosis of “suspected malaria infection” at the Ministry of Heath Regional Hospital in Bamenda, Cameroon. Patients were referred for phlebotomy if they exhibited the following clinical signs and symptoms of a malarial infection: fever (temperature > 38.0 °C) with associated arthralgia, myalgia, headache, abdominal pain, and malaise, or recent diagnosis of malaria within the past 6 weeks. No changes occurred in the standard of care prior to the study. The blood specimens were collected and slides were prepared for thin and thick smears, de-identified, and assigned a sequential identification number. Two additional sets of thin and thick blood smears were made for each patient and a drop of blood was collected on FTA™ filter paper cards (Whatman International Ltd., Maidstone, England) to be used for confirmatory PCR analysis in our research laboratory in the United States. Positive controls from malaria-positive participants were prepared for further standardization and training.

### Microscopy assay

**Fig. 1:**
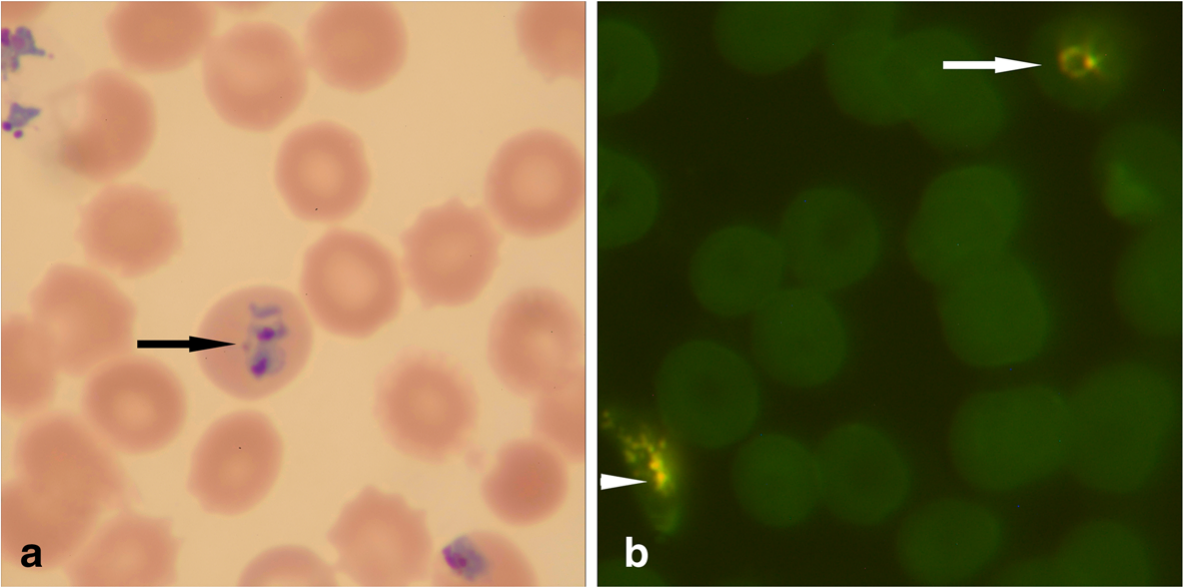
Comparison of microscopy methods. Panel **a** displays a positive sample using GS showing a positive red cell by the black arrow. Panel **b** is an example of a positive sample using the FM system. The white arrow indicates a trophozoite while the arrowhead indicates a schizont. All images are at 1000 X magnification

The first set of thin and thick smears were stained with the FAST Malaria stain kit (427760, QBC Diagnostics Inc., Port Matilda, PA) and evaluated with the ParaLens Advance^TM^ microscope attachment (QBC Diagnostics Inc., Port Matilda, PA) at 1000X magnification. The stain is proprietary and developed specifically to enhance *Plasmodium* species and the microscope LED attachment can be charged using sunlight. The second set of thin and thick smears were stained with the traditional Giemsa stain (Spectrum Chemicals, Gardena, CA) and evaluated with bright field microscopy for the presence of parasites. Thick smears were used for screening first at 1000X magnification. If parasites were detected using the thick smears, there was confirmatory examination of the thin smears at 1000X to detect parasites and life cycle stage. These two sets of samples were randomized to prevent sequential reading of the same sample by the same technologist. The samples were divided randomly into three unique sets, which were read blind by three different technicians with differing training and experience levels (technicians A, B, and C). There was no cross examination of samples between the technicians. Additionally, a randomly selected set of samples was analysed blind by a highly skilled technician (technician D) using the GS and FM system at the Mezam Polyclinic HIV/AIDS Treatment Center. To minimize any variability due to potential fluorescence stain fading, all slides stained with FM were read within 48 h of staining. All parties were blinded to the results of the microscopy studies and PCR analysis that was carried out on a later date. Figure 1 demonstrates the microscopic difference between both techniques.

### Polymerase chain reaction

A two-step nested PCR assay was used to confirm the results of the microscopy studies. PCR has been shown to have high sensitivity in the detection of malarial species and was, therefore, chosen to compare the microscopy results [24, 25]. This high level of accuracy was the basis for our utilization of the technique for validation of results. Oligonucleotide primers were chosen to detect a highly conserved region of the small subunit ribosomal RNA genes for the genus *Plasmodium*.

The assay was performed directly from the blood samples preserved on the FTA^TM^ filter paper. A 1 mm punch was taken from the filter paper and washed in 150 μL nuclease- free water for 5 min at room temperature. The PCR assay was performed within 3 h of washing. The first nest reaction was performed in a 50 μL reaction vessel using a Phusion Blood Direct PCR Kit (Thermo Scientific, Waltham, MA) and the primers rPLU1 and rPLU5, which produce a 1 640 bp amplicon and serve as the template DNA for the second nest reaction. The second nest was performed at a total reaction volume of 25 μL using a Phusion^®^ High-Fidelity DNA Polymerase kit (New England Biolabs, Ipswich, MA) and the primers rPLU3 and rPLU4, which produced a 240 bp amplicon. Previous studies have shown reliable results using these primer sets [26–28].

### Statistical analysis

Four technicians analysed the blood smears from 499 patients. The accuracy measures from each technician for both GS and FM including PPV, NPV, sensitivity, specificity, and error rate are summarized. Descriptive statistics such as mean and standard deviation were obtained. A one-tail proportional z-score test was employed to determine whether PPV, NPV, and error rates produced by FM were significantly lower than GS. A *P* < 0.05 was considered to indicate a statistical significance. Statistical Package for the Social Science (SPSS) version 23 (IBM SPSS Statistics, Chicago, IL) was used for the analysis.

## Results

There were 522 patients with a presumptive diagnosis of malaria presented for the study and all provided blood samples. The ages ranged between 1 month to 92 years with a mean age of 35.35 years (SD = 19.44). Of the 522 samples, 23 were excluded from the final analysis due to either poor staining (20 slides), damage (2 slides were accidentally broken), or mislabelling (1 with unclear label). Two patients were excluded due to concomitant lymphoma, which could not be excluded as the source of the fever. In this study, 469 patients received therapy irrespective of the laboratory diagnosis (the PCR results were not known at that time). After final analysis, 30 samples (6.01%) were positive via PCR as determined by a 240 bp region corresponding to our primer length. Five of the 30 positive samples (16.67%) were specimens from individuals under the age of 18. Given that *Plasmodium falciparum* is indigenous to this area and the fact that all samples confirmed by PCR were reviewed by two clinical pathologists and determined to be *P. falciparum* based upon microscopy, it was not considered cost-effective to further validate the species by additional PCR. To our knowledge, *P. vivax*, *P. ovale,* and *P. malariae* have not been identified in this area of Cameroon, as in the South Western coastal region where more than 85% are *P. falciparum* [2, 5]. Comparison of the PCR results with the microscopy data obtained from our clinic in Cameroon revealed a high level of discrepancy between each of the three technicians using the GS thick and thin smears. In contrast, more consistency in diagnostic accuracy was observed with FM, regardless of technician skill level, when using the PCR results as the diagnostic standard. For the GS samples, there were 35 false positive and 22 false negative results, while 18 false positive and 22 false negative results were obtained with FM. Proportional z-score test indicated that the error rate of GS (error rate = 0.1169) was significantly higher than that of FM (error rate = 0.0802) with *p* = 0.0259 (z = 1.95). Both one- and two-tailed proportional z-score tests were applied showing significance, though due to our prior publication for a similar study using one-tail z-score, we chose to use this for reporting statistical significance [18].

Using the PCR analysis to confirm the results using each method, the PPV and NPV were determined for both methods. The mean of PPV from Giemsa (19.62%) and FM (27.99%) methods were significantly different (z = −3.10, *P* = 0.001). There was no significant difference of NPV between GS (95.01%) and FM (95.28%) methods (z = 0.1984, *P* = 0.42). Both microscopy methods had identical sensitivities (26.67%); however, there was an improved specificity using the FM method (96.21% vs. 92.78% with GS, *P =* 0.046, z = 1.6826). The data, including individual technician breakdowns, are summarized in Table 1.

**Table 1:** Summary of the microscopy findings for GS and FM

		GS					FM				
Technician^a^	N^b^	PPV, %	NPV, %	Errors^c^, %	Sen, %	Spec, %	PPV, %	NPV, %	Errors, %	Sen, %	Spec, %
A	138	13.33	92.59	24.64	33.33	79.37	33.33	93.80	8.70	38.46	92.37
B	226	26.67	96.17	8.41	33.33	94.81	23.08	96.19	7.96	27.27	95.28
C	138	0.00	94.85	6.52	0.00	98.47	0.00	94.66	10.14	0	94.66
D	218	38.46	96.41	7.21	41.67	95.92	55.56	96.48	5.29	41.67	97.96
Mean		19.62	95.01	11.69	27.08	92.14	27.99	95.28	8.02	26.85	95.07
SD		16.62	1.75	8.66	18.48	8.65	23.07	1.27	2.04	18.93	2.30

^a^ Technicians ordered with increasing experience and training level

^b^ The total patient number was 499, however three samples were analysed by two technicians yielding the same results leading to a total number of reads of 501

^c^ Error rate refers to the combination of the false positive and false negative readings in a percentage format

## Discussion

Laboratory accuracy is a necessity in the diagnosis of malaria, as there is currently a high incidence of misdiagnosis leading to unnecessary or non-beneficial treatment of other infections [7, 8, 29]. Studies in Africa suggests that 50% to 99% of antimalarial agents are prescribed to patients who are unlikely to have malaria [30]. This is especially important in this region of the northwest province of Cameroon where the prevalence appears to be relatively low compared to the low-lying coastal regions. We were surprised at the low number of malaria-positive samples based upon our impression from the number of patients seen in previous years, although this could have been due to seasonal variation (less rain). The year of the study, 2011, was notable for less rain and generally fewer cases of “suspected malaria” presenting at the Regional Hospital and fewer confirmed malaria cases. Table 2 illustrates the number of patients with confirmed cases of malaria between 2009 and 2013.

**Table 2:** Confirmed and treated malaria cases at Bamenda Regional Hospital between 2009 and 2013

	Number of cases				
	2009	2010	2011	2012	2013
January	614	513	452	400	585
February	539	508	523	539	425
March	365	321	324	463	482
April	426	492	475	388	500
May	518	498	426	338	342
June	436	559	435	387	362
July	485	430	484	359	301
August	307	481	322	266	273
September	304	503	596	287	447
October	435	731	637	609	376
November	301	683	514	330	568
December	365	480	400	356	425
Total	5 095	6 199	5 588	4 722	5 086

The WHO has expressed concern over the lack of quality laboratory diagnostic tests in regions where malaria is endemic, which leads to increased financial burden and waste of resources [3]. Whether diagnosis is empirical or by laboratory confirmation in the form of microscopy, there is a high level of error, especially with the use of non-accredited laboratories. Anecdotally, after interviewing clinicians involved during and after our study, we found that there is a wide range of clinical symptoms other than fever to make the diagnosis of malaria. The low specificity of clinical diagnosis, combined with the insensitive diagnostic tools available, makes this a problematic method in the treatment and management of individuals afflicted by malaria or related diseases.

There are many factors affecting the laboratory diagnosis of malaria using traditional methods that must be taken into consideration when interpreting a diagnostic report. In the case of GS, lack of appropriate resources and training are a key feature [31, 32]. Additionally, early infections may be associated with false negative results due to low numbers of parasites in the blood, which can be overlooked [31]. Further, the accuracy of the GS method as well as alternative microscopy tests may also be affected by the species of parasite and the stage of the life cycle [33]. Field studies using microscopy suffer from reduced accuracy and yield low sensitivity making laboratory diagnosis unreliable and impacting clinician confidence [7, 34]. The current study was prompted by the need to determine how frequently malaria was responsible for fever in our sample population, as well as the anecdotal lack of reliability and accessibility of the RDTs available in the hospital in 2011. There is an urgent need for a reliable, validated, inexpensive RDT for malaria to replace more cumbersome techniques. In the interim, the WHO and the Foundation for Innovative New Diagnostics have emphasized the importance of a rapid point of care (POC) test for malaria and the WHO offers valuable guidance on how to validate RDTs for local use [4]. At the time of this writing, there are no validated POC RDTs for malaria diagnosis with FDA approval or CE Marking recommended by the WHO.

The detection threshold for GS thick blood smears increases with decreasing technician skill level and quality of equipment [35]. Even with common equipment and training, as we found, there is discrepancy in diagnosis between different laboratories leading to decreased inter-examiner reliability [36, 37]. Further, specificity of the test is variable, especially in local laboratories where training and resources may be inadequate. This may arise from improper preparation of the slides, as well as the presence of other organisms or cells appearing similar to *Plasmodium* species [38].

Our data indicate an increase in the PPV and specificity in the diagnosis of malaria using FM. We found that FM was faster than GS and that there was significantly less artefactual staining, which could explain why some of the technicians were able to recognize the parasites more easily. Our data suggest this increased specificity is less sensitive to the length of training. The latter finding is important in addressing problems associated with the diagnosis of malaria, as the level of technician training is highly correlated with the use of GS. However, given the low number of confirmed malaria cases in this study, it is important to evaluate the impact of skill and training on the GS method in an area with a much higher incidence of malaria since conventional staining techniques such as GM and FM are likely to be in use for long into the future. While technician concordance was not assessed in this study, there appeared to be increased reliability with FM when compared with the PCR results. This improved accuracy despite variability in technician experience using the FM system, if substantiated, could yield improvement in laboratory testing, reduce cost, and reduce the misuse of medications leading to increased drug resistance. Employing FM technique to increase the PPV and specificity in the detection of a *Plasmodium* infection could enhance clinician confidence in laboratory data and reduce reliance on clinical symptomatology, which is inherently nonspecific [7–9].

Interestingly, despite the increased sensitivity of FM in the diagnosis of tuberculosis in a similar format, this was not observed for malaria in this study [23]. We postulate this is due to the small number of infected samples and lack of statistical power to assess for increased sensitivity. As previously described, only 30 samples (6.01%) were positive via PCR and there were equal numbers of true positives in both sample sets. This may be coincidental and a larger pool of true positive samples would be necessary to ascertain differences in sensitivity. Nonetheless, FM shows sensitivity at least equal to that obtained by GS (26.67%) with the potential of less reliance on technician skill level.

While studies have suggested a prevalence of malaria in Cameroon ranging from 17.2% to 53.21% over the past 9 years [1], there are large differences between the coastal areas such as Buea and the high grasslands further north which varies from year to year depending upon rainfall. The WHO estimated an incidence of malaria around 25% for the country as a whole in 2015 [5]. Despite this, we found a PCR-positive rate of only 6.01%, lending support for our hypothesis that malaria is over-diagnosed and that antimalarial medications are over-utilized. While technological error may also have contributed to this finding, this is unlikely since our assay was robust with positive and negative controls included to assess the accuracy of the PCR assay for each patient sample replicated. Further, this low positive rate confirmed by PCR was within the range of that seen via microscopy (9.42% for GS and 6.01% for FM). It is likely that patients negative for a diagnosis of malaria had unrelated febrile bacterial or viral illnesses, perhaps associated with HIV [8, 9]. Furthermore, despite the low number of confirmed positive cases, 469 patients were treated with antimalarial agents. These patients unnecessarily received treatment because they were believed to have the disease. This further corroborates the lack of sensitivity with subjective signs and symptoms of malaria and highlights the need for more accurate diagnosis.

Anecdotally, physicians were reluctant not to treat all patients symptomatically before results were known, even with the negative test results. Despite this, the preliminary findings in our study led to a policy change at the Cameroon Ministry of Heath Regional Hospital in Bamenda in an effort to mitigate these unnecessary treatments. However, as shown elsewhere, physicians and allied health personnel practices are resistant to change [9]. This policy change to promote the use of microscopy or a validated RDT in place of empirical treatment of malaria has been implemented following results of this study but has proved very difficult to enforce as of July 2016.

## Conclusions

This study showed that there was a low number of malaria-positive patients of those presenting with a presumptive diagnosis of malaria at the Bamenda Regional Hospital in 2011. We also showed a correlation between technician training and the accuracy of malaria diagnosis using bright field microscopy with GS. Differences in skill level diminished when using the FM system, which is faster, slightly more specific, had an increased PPV, and reduced staining artifact compared to standard GS. However, a significantly low sensitivity of both GS and FM was present even with highly experienced technicians. This study highlights the need for an inexpensive diagnostic method with improved reliability and acknowledges the potential for emergence of drug resistance exacerbated by the widespread practice of empirical treatment for malaria. Public health systems should be vigilant to encourage improvement in physician prescribing practices. Continued education of technical personnel is important, as is encouragement in the use of validated RDTs when resources are available. Finally, education of the public as to the causes of drug resistance enhanced by self-medication for malaria is important. A larger study to determine and compare the impact of technical expertise using the two methods in an area with a higher prevalence of malaria is planned with the additional objective to compare these methods against available RDTs and validate our current findings. Furthermore, it would be worth assessing the concordance in diagnostic accuracy between technicians of varying skill levels on the same samples.

## Additional file

Additional file 1: Multilingual abstracts in the five official working languages of the United Nations. (PDF 816 kb)

## Abbreviations

FAST: Fluorescence and Staining Technologies
FM: QBC Diagnostics’ FAST Malaria stain
GS: Giemsa stain
IRB: Institutional review board
NPV: Negative predictive value
PCR: Polymerase chain reaction
POC: Point of care
PPV: Positive predictive value
RDT: Rapid diagnostic test
WHO: World Health Organization

## References

[1] Ndong IC, van Reenen M, Boakye DA, Mbacham WF, Grobler AF (2014). Trends in malaria admissions at the Mbakong Health Centre of the North West Region of Cameroon: a retrospective study. Malar J.

[2] Bigoga JD, Manga L, Titanji VPK, Coetzee M, Leke RGF (2007). Malaria vectors and transmission dynamics in coastal south-western Cameroon. Malar J.

[3] World Health Organization (2013). World malaria report: 2013.

[4] World Health Organization (2015). Guidelines for the treatment of malaria.

[5] World Health Organization (2015). World malaria report: 2015.

[6] Sundararajan R, Mwanga-Amumpaire J, Adrama H, Tumuhairwe J, Mbabazi S, Mworozi K, Carroll R, Bangsberg D, Boum Ii Y, Ware NC (2015). Sociocultural and structural factors contributing to delays in treatment for children with severe malaria: A qualitative study in southwestern Uganda. Am J Trop Med Hyg.

[7] Mwangi TW, Mohammed M, Dayo H, Snow RW, Marsh K (2005). Clinical algorithms for malaria diagnosis lack utility among people of different age groups. Trop Med Int Health.

[8] Rakotonirina H, Barnadas C, Raherijafy R, Andrianantenaina H, Ratsimbasoa A, Randrianasolo L, Jahevitra M, Andriantsoanirina V, Ménard D (2008). Accuracy and reliability of malaria diagnostic techniques for guiding febrile outpatient treatment in malaria-endemic countries. Am J Trop Med Hyg.

[9] Reyburn H, Mbakilwa H, Mwangi R, Mwerinde O, Olomi R, Drakeley C, Whitty CJM (2007). Rapid diagnostic tests compared with malaria microscopy for guiding outpatient treatment of febrile illness in Tanzania: randomised trial. Br Med J.

[10] Bloland PB, Kachur SP, Williams HA (2003). Trends in antimalarial drug deployment in sub-Saharan Africa. J Exp Biol.

[11] Bell D, Wongsrichanalai C, Barnwell JW (2006). Ensuring quality and access for malaria diagnosis: how can it be achieved?. Nat Rev Microbiol.

[12] White NJ (2004). Antimalarial drug resistance. J Clin Invest.

[13] Stauffer W, Fischer PR (2003). Diagnosis and treatment of malaria in children. Clin Infect Dis.

[14] D'Acremont V, Kilowoko M, Kyungu E, Philipina S, Sangu W, Kahama-Maro J, Lengeler C, Cherpillod P, Kaiser L, Genton B (2014). Beyond malaria—causes of fever in outpatient Tanzanian children. N Engl J Med.

[15] World Health Organization (2009). Malaria microscopy quality assurance manual, version 1.

[16] Wongsrichanalai C, Barcus MJ, Muth S, Sutamihardja A, Wernsdorfer WH (2007). A review of malaria diagnostic tools: microscopy and rapid diagnostic test (RDT). Am J Trop Med Hyg.

[17] Schroeder LF, Amukele T (2014). Medical laboratories in Sub-Saharan Africa that meet international quality standards. Am J Clin Pathol.

[18] Lema C, Dionne K, Ayuk L, Awasom C, Sander M, McArthur C, Achu P, Parrish N (2013). Evaluation of the ParaLens LED microscope attachment versus standard fluorescence microscopy for detection of *Mycobacteria*. J Tuberc Res.

[19] Gay F, Traoré B, Zanoni J, Danis M, Fribourg-Blanc A (1996). Direct acridine orange fluorescence examination of blood slides compared to current techniques for malaria diagnosis. Trans R Soc Trop Med Hyg.

[20] Kawamoto F (1991). Rapid diagnosis of malaria by fluorescence microscopy with light microscope and interference filter. Lancet.

[21] Makler MT, Palmer CJ, Ager AL (1998). A review of practical techniques for the diagnosis of malaria. Ann Trop Med Parasitol.

[22] Kuhn W, Armstrong D, Atteberry S, Dewbrey E, Smith D, Hooper N (2010). Usefulness of the Paralens™ fluorescent microscope adaptor for the identification of mycobacteria in both field and laboratory settings. Open Microbiol J.

[23] Cuevas LE, Al-Sonboli N, Lawson L, Yassin MA, Arbide I, Al-Aghbari N, Bahadur Sherchand J, Al-Absi A, Emenyonu EN, Merid Y (2011). LED fluorescence microscopy for the diagnosis of pulmonary tuberculosis: a multi-country cross-sectional evaluation. PLoS Med.

[24] Snounou G, Singh B (2002). Nested PCR analysis of Plasmodium parasites. Methods Mol Med.

[25] Snounou G, Viriyakosol S, Zhu XP, Jarra W, Pinheiro L, do Rosario VE, Thaithong S, Brown KN (1993). High sensitivity of detection of human malaria parasites by the use of nested polymerase chain reaction. Mol Biochem Parasitol.

[26] Fuehrer H-P, Fally MA, Habler VE, Starzengruber P, Swoboda P, Noedl H (2011). Novel nested direct PCR technique for malaria diagnosis using filter paper samples. J Clin Microbiol.

[27] Fuehrer H-P, Stadler M-T, Buczolich K, Bloeschl I, Noedl H (2012). Two techniques to simultaneously identify Plasmodium ovale curtisi and P. ovale wallikeri using the small subunit rRNA gene. J Clin Microbiol.

[28] Singh B, Bobogare A, Cox-Singh J, Snounou G, Abdullah MS, Rahman HA (1999). A genus- and species-specific nested polymerase chain reaction malaria detection assay for epidemiologic studies. Am J Trop Med Hyg.

[29] Källander K, Nsungwa-Sabiiti J, Peterson S (2004). Symptom overlap for malaria and pneumonia—policy implications for home management strategies. Acta Trop.

[30] Amexo M, Tolhurst R, Barnish G, Bates I (2004). Malaria misdiagnosis: effects on the poor and vulnerable. Lancet.

[31] Kain KC, Harrington MA, Tennyson S, Keystone JS (1998). Imported malaria: Prospective analysis of problems in diagnosis and management. Clin Infect Dis.

[32] Payne D (1988). Use and limitations of light microscopy for diagnosing malaria at the primary health care level. Bull World Health Organ.

[33] Milne LM, Kyi MS, Chiodini PL, Warhurst DC (1994). Accuracy of routine laboratory diagnosis of malaria in the United Kingdom. J Clin Pathol.

[34] Ochola LB, Vounatsou P, Smith T, Mabaso MLH, Newton C (2006). The reliability of diagnostic techniques in the diagnosis and management of malaria in the absence of a gold standard. Lancet Infect Dis.

[35] Wongsrichanalai C, Pickard AL, Wernsdorfer WH, Meshnick SR (2002). Epidemiology of drug-resistant malaria. Lancet Infect Dis.

[36] Durrhelm DN, Becker PJ, Billinghurst K, Brink A (1997). Diagnostic disagreement--the lessons learnt from malaria diagnosis in Mpumalanga. S Afr Med J.

[37] Maguire JD, Lederman ER, Barcus MJ, Ap O'Meara W, Jordon RG, Duong S, Muth S, Sismadi P, Bangs MJ, Prescott WR (2006). Production and validation of durable, high quality standardized malaria microscopy slides for teaching, testing and quality assurance during an era of declining diagnostic proficiency. Malar J.

[38] McKenzie FE, Sirichaisinthop J, Miller RS, Gasser RA, Wongsrichanalai C (2003). Dependence of malaria detection and species diagnosis by microscopy on parasite density. Am J Trop Med Hyg.

