# Supplementary material for: Malaria over-diagnosis in Cameroon: diagnostic accuracy of Fluorescence and Staining Technologies (FAST) Malaria Stain and LED microscopy versus Giemsa and bright field microscopy validated by polymerase chain reaction
Source: Infect Dis Poverty. 2017 Apr 4;6:32. doi: 10.1186/s40249-017-0251-0 (PMC5379548; doi:10.1186/s40249-017-0251-0)
Supplement: Additional file 1: — Multilingual abstracts in the five official working languages of the United Nations. (PDF 816 kb) [file 40249_2017_251_MOESM1_ESM.pdf]

Translation of the abstract into the five official working languages of the United Nations

الإفراط في تشخيص مرض الملاريا في الكاميرون: التأكد من دقة التشخيص باستخدام تقنيات العلامات اللونية والفلورسنت لعينة الملاريا و الصمام المجهري الباعث للضوء مقابل ملوّن غيمزا والفحص المجهري بالسّاحة السّاطعة من خلال تفاعل البوليميراز المتسلسل.

شون ام بارسل، ستيفن إي غاستافسون، إدوارد فريدلاندر، الكساندر إي شنيرا، أداروسوي جي أديغبولو، ينغ لوي، نيكول إم باريش، سيد جمال، إيف لوفتاس، ليو أيوك، تشارلز أواسوم، كارولين جي هنري، كارول بي ماك أرثر.

ملخص الدراسة

خلفية الموضوع: تعد الملاريا مشكلة صحية عالمية رئيسية ويعود هذا العبئ المستمر لهذا المرض بشكل جزئي إلى الصعوبات في التشخيص. توصي منظمة الصحة العالمية بإجراء الإختبار التأكيدي بستخدام التقنيات التي تعتمد على التشخيص المجهري أو الاختبارات التشخيصية السريعة (RDT) لجميع الحالات المشتبه اصابتها بمرض الملاريا. هناك مسببات متعددة للحمى في المناطق التي يتأصل فيها سلالة بلازموديوم و هو ماقد يؤدي إلى التشخيص الخاطئ للمرض وخصوصاً في المناطق التي ينتشر بين سكانها مرض الإيدز وعند الأطفال. ولتحديد وتيرة الإصابة بعدوى مرض الملاريا عند المرضى المصابين بالحمى خلال فترة 8 أشهر في المستشفى الإقليمي في بامندا في الكاميرون قمنا بتقييم الفعالية السريرية لتقنية العلامات اللونية والفلوريسنت (F.A.S.T.) لعينة الملاريا و نظام البارالينز ادفانس المجهري (FM) مقارنة مع الفحص المجهري التقليدي بالساحة الساطعة و صبغة غيمزا (GS).

منهجية البحث: تم تقيم عيّنات الدم الطرفية التي تم أخذها من 522 مريض يشتبه بإصابتهم بالملاريا حسب التشخيص السريري باستخدام المجهر الفلوري وصبغة غيمزا. كان تفاعل البوليميراز المتسلسل المتداخل هو القاعدة الذهبية للمقارنة بين الطريقتين. تم تحديد ايجابية تفاعل الوليميراز المتسلسل والحساسية والدقة والقيمة المتنبّأة الإيجابية والقيمة المتنبّأة السلبية.

النتائج: تم ادراج 499 عينة في التحليل النهائي. كانت نتيجة 30 من هذه العينات ايجابية عبر تفاعل البوليميراز المتسلسل (6.01%) بمتوسط القيمة المتنبّأة الإيجابية 19.62% و 27.99% لصبغة غيمزا و المجهر الفلوري على التوالي. وكانت القيمة السلبية المتنبّأة 95.01% و 95.28% لصبغة غيمزا و المجهر الفلوري على التوالي. نسبة الحساسية كانت 26.67% في كلا المجموعتين وكانت الدقة 92.78% و 96.21% لصبغة غيمزا و المجهر الفلوري على التوالي. وقد لوحظ زيادة مستوى التباين بين الفنيين بناءاً على مستوى المهارة في استخدام صبغة غيمزا والتي لم يتم ملاحظتها في المجهر الفلوري FM .

الخاتمة: تبين أن تردد عدوى الملاريا التي تم تأكيدها عبر تفاعل البوليميراز المتسلسل بين المرضى الذين يعانون من الحمى وأعراض أخرى لمرض الملاريا كان أقل بشكل كبير من المتوقع بناءا على الإشتباه بها من قبل الأطباء خلال الفحص السريري. ولوحظ وجود ارتباط بين مهارة الفني ودقة تشخيص مرض الملاريا باستخدام صبغة غيمزا والتي كانت أقل وضوحا بالإضافة إلى ذلك فقد ازدادت دقة التشخيص باستخدام الفحص المجهري الفلوري وتم FM. بستخدام المجهر الفلوري تحسين القيمة المتنبّأة الإيجابية مما يشير إلى أن هذا النهج منخفض التكلفة قد يكون مفيداً في البيئة ذات الموارد المحدودة. حسب الرويات التي يتم تناقلها فإن الأطباء يترددون بعدم علاج كافة المرضى حسب الأعراض قبل معرفة نتائج التحليل على الرغم من التشخيص المجهري الذي اعطى نتيجة سلبية لوجود المرض وهو مايسلط الضوء على الحاجة الى تقديم مزيد من التعليم للأطباء لتجنب مثل هذه الممارسات التي تتضمن تقديم الرعاية الصحية الغير ضرورية. ويجري التخطيط لدراسة أكبر في منطقة ذات الانتشار الواسع المعروف لطريقتي الفحص المجهري مقارنة بالإختبارات التشخيصية السريعة.

Translated from English version into Arabic by Mahmoud Sami, through

TRANSLATORS
WITHOUT BORDERS

喀麦隆疟疾过度诊断：通过聚合酶链式反应验证荧光染色技术 LED 镜检和吉氏染色光学显微镜诊断的准确性

Sean M. Parsel, Steven A. Gustafson, Edward Friedlander, Alexander A. Shnyra, Aderosoye J. Adegbulu, Ying Liu, Nicole M. Parrish, Syed A. Jamal, Eve Lofthus, Leo Ayuk, Charles Awasom, Carolyn J. Henry, Carole P. McArthur

摘要

引言：疟疾是主要的全球公共卫生问题之一，所造成的持续疾病负担有一部分是因疾病诊断困难所致。世界卫生组织建议采用镜检和快速诊断试剂（RDT）对所有疑似病例进行确诊。在具有本地疟原虫虫种的地区，有多种发热病因会引起误诊，特别是在 HIV 流行的人群和儿童中。为了确认喀麦隆巴门达地区医院 8 个月中发热病人的疟疾感染率，我们评估了荧光染色技术(F.A.S.T.)和 ParaLens Advance™ 镜检系统的临床诊断效能，并与传统的镜检法和吉氏染色结果进行比较。

方法：采集临床诊断的 522 例疑似病例的外周血，经吉氏染色(GS)和 ParaLens Advance™ 镜检系统(FM)方法进行评估。以巢式 PCR 作为金标准对两种方法进行比较，并计算 PCR 阳性率、敏感性、特异性、阳性预测值(PPV)和阴性预测值(NPV)。

结果：最终有 499 份样本纳入分析。其中，30 份经 PCR 检测为阳性(6.01%)，GS 和 FM 的平均PPV分别为19.62%和27.99%，平均NPV分别为95.01% 和 95.28%，敏感性均为26.67%，特异性分别为 92.78%和 96.21%。检测人员的 GS 技术水平的高低会导致诊断结果的差异，但是采用 FM 不受影响。

结论：在有发热和其他疟疾症状的患者中，经 PCR 证实的疟疾病例数远低于医生临床鉴定的疑似病例。吉氏染色镜检的技术水平和诊断准确性有一定相关性，但采用 FM 时，这种关系并不非常显著。此外，FM 的特异性较高，并且改善了 PPV，表明低成本的方法可能更适用于资源有限的环境。有趣的是，医生在诊断结果未知的情况下，不愿意对患者进行对症治疗，尽管有一些阴性镜检诊断结果，这也进一步表明仍需要对医生进行教育培训来避免过度治疗。我们计划在已知疟疾重度流行区对这两种镜检方法和 RDT 的检测效能进行比较。

Translated from English version into Chinese by Xin-Yu Feng, edited by Pin Yang, through

CDC CHINESE CENTER FOR DISEASE CONTROL AND PREVENTION
NATIONAL INSTITUTE OF PARASITIC DISEASES

Surdiagnostic du paludisme au Cameroun : précision diagnostique des méthodes par fluorescence et coloration et de la microscopie à LED par rapport à la coloration de Giemsa et à la microscopie en champ clair, validée par une réaction en chaîne à la polymérase.

Sean M. Parsel, Steven A. Gustafson, Edward Friedlander, Alexander A. Shnyra, Aderosoye J. Adegbulu, Ying Liu, Nicole M. Parrish, Syed A. Jamal, Eve Lofthus, Leo Ayuk, Charles Awasom, Carolyn J. Henry, Carole P. McArthur

Résumé

Contexte : Le paludisme est un problème majeur de santé publique dont la persistance est due, en partie, à des difficultés de diagnostic. L'Organisation Mondiale de la Santé préconise des tests de confirmation par microscopie ou des tests de diagnostic rapide (TDR) pour tous les cas suspects de paludisme. Dans les zones de présence de *Plasmodium*, les fièvres peuvent avoir de nombreuses

autres causes et il en résulte des erreurs de diagnostic, notamment dans les populations également affectées par le VIH et chez les enfants. Afin de déterminer la fréquence des infections palustres chez des patients fébriles reçus à l'Hôpital régional de Bamenda au Cameroun, nous avons évalué l'efficacité clinique de la coloration spécifique FAST (Fluorescence and Staining Technology) et du système de microscopie en fluorescence (MF) ParaLens Advance™ et l'avons comparée à celle de la microscopie en champ clair et de la coloration de Giemsa traditionnelles.

Méthodes : Des échantillons de sang périphérique ont été prélevés sur 522 patients suspectés d'être des cas de paludisme et examinés par coloration de Giemsa et microscopie MF. Une RCP emboîtée constituait la référence pour la comparaison des deux méthodes. La positivité à la RCP, la sensibilité, la spécificité, ainsi que la valeur prédictive positive (VPP) et négative (VPN) ont été déterminées.

Résultats : L'analyse finale porte sur 499 échantillons. Parmi ceux-ci, 30 étaient positifs à la RCP (6,01 %), avec une VPP moyenne de 19,62 % pour la coloration de Giemsa et 27,99 % pour la microscopie MF. La VPN moyenne était de 95,01 % pour la coloration de Giemsa et 95,28 % pour la microscopie MF. La sensibilité était de 26,67 % dans les deux groupes et la spécificité de 92,78 % pour la coloration de Giemsa et 96,21 % pour la microscopie MF. Des écarts de diagnostics entre les techniciens en fonction de leur habileté ont été constatés pour la coloration de Giemsa mais pas pour la microscopie MF.

Conclusions : La fréquence des infections palustres confirmées par la RCP parmi les patients fiévreux et présentant d'autres symptômes du paludisme était très inférieure à celle attendue sur la base des soupçons cliniques des médecins. Une corrélation entre les compétences des techniciens et la précision du diagnostic de paludisme a été constatée avec la coloration de Giemsa mais moins avec la microscopie en fluorescence. En outre, la microscopie MF avait une spécificité et une VPP supérieures, ce qui suggère que cette approche relativement peu coûteuse pourrait être utile lorsque les ressources sont limitées. Pour l'anecdote, les médecins se sont montrés réticents à s'abstenir de donner un traitement symptomatique aux patients avant que les résultats soient connus et même lorsque le diagnostic de la microscopie était négatif, ce qui met en lumière la nécessité d'une éducation plus poussée du corps médical afin d'éviter les surtraitements. Une étude plus vaste, dans une zone où la prévalence du paludisme est notoirement élevée, est à l'étude afin de comparer les deux méthodes de microscopie par rapport aux TDR rapides.

Translated from English version into French by Suzanne Assenat, through

TRANSLATORS
WITHOUT BORDERS

Гипердиагностика малярии в Камеруне: диагностическая точность технологии флуоресцентного окрашивания на малярию (F.A.S.T.) и светодиодной микроскопии в сравнении с красителем Гимзы и светлопольной микроскопией, проверенная по методу полимерной цепной реакции.

Шон М. Парсел (Sean M. Parsel), Стивен А. Густафсон (Steven A. Gustafson), Эдвард Фридландер (Edward Friedlander), Александр А. Шныра (Alexander A. Shnyra), Адеросойе Дж.

Адегбулу (Aderosoye J. Adegbulu), Ин Лю (Ying Liu), Николь М. Парриш (Nicole M. Parrish), Саид А. Джамал (Syed A. Jamal), Ева Лофтус (Eve Lofthus), Лео Аюк (Leo Ayuk), Чарльз Авасом (Charles Awasom), Кэролин Дж. Генри (Carolyn J. Henry), Кэрол П. МакАртур (Carole P. McArthur)

Аннотация

Краткое описание. Малярия представляет собой серьёзную проблему для здравоохранения во всем мире. Продолжающееся бремя малярии частично связано с трудностями диагностирования болезни. Всемирная организация здравоохранения рекомендует подтверждающую проверку во всех случаях подозрения на малярию при помощи методов микроскопии или быстрых диагностических тестов. В регионах, где виды *Plasmodium* являются местными, существует множество причин для лихорадки, что приводит в неправильной постановке диагноза, особенно среди детей и слоёв населения, где широко распространена ВИЧ-инфекция. Для установления частоты заболевания малярией у лихорадящих больных в региональном госпитале в г. Баменда (Камерун) мы в течение 8 месяцев оценивали медицинскую эффективность технологии флуоресцентного окрашивания на малярию (F.A.S.T.) и системы ParaLens Advance™ (ФМ) и сравнивали её с эффективностью традиционной светлопольной микроскопии и красителя Гимзы (МГ).

Методы. Образцы периферической крови 522 больных с клиническим диагнозом «подозрение на малярию» были исследованы с использованием методов МГ и ФМ. В качестве эталона для сравнения этих двух методов применялась гнездовая полимерная цепная реакция (ПЦР). Были определены положительность ПЦР, чувствительность, специфичность, положительная прогностическая значимость (ППЗ) и отрицательная прогностическая значимость (ОПЗ).

Результаты. В окончательный анализ были включены 499 образцов. Из них 30 были положительными по ПЦР (6,01%) со средним ППЗ 19,62% и 27,99% для МГ и ФМ соответственно. Средняя ОПЗ была 95,01% и 95,28% для МГ и ФМ соответственно. Чувствительность составила 26,67% в обеих группах, и специфичность была 92,78% и 96,21% для МГ и ФМ соответственно. Наблюдалось повышенное число расхождений в диагностике у лаборантов, применявших МГ, в зависимости от уровня их квалификации, что не было замечено в отношении ФМ.

Выводы. Проверенная методом ПЦР, заражённость малярией у больных с лихорадкой и другими симптомами малярии была существенно ниже, чем предполагалось на основании клинических подозрений врачей. Была отмечена зависимость точности диагностирования малярии от квалификации лаборантов при применении МГ, которая была менее выражена при применении ФМ. Кроме того, метод ФМ повысил специфичность и улучшил ППЗ, и это позволяет предположить, что этот относительно низкозатратный подход может быть использован в средах с ограниченными ресурсами. Как показал опыт, врачи неохотно отказываются от лечения всех больных симптоматически до того, как известны результаты анализов, и, несмотря на отрицательный тест микроскопии, что подчёркивает необходимость дальнейшего обучения врачей для того, чтобы избежать излишнего лечения. В настоящее время планируется проведение более широкого исследования в районе с известным высоким уровнем заболеваемости для сравнения двух методов микроскопии с имеющимися быстрыми диагностическими тестами.

Translated from English version into Russian by Natalia Potashnik, through

TRANSLATORS WITHOUT BORDERS

Sobrediagnóstico del paludismo en Camerún: exactitud del diagnóstico de tinción para paludismo con la Tecnología de Tinción y Fluorescencia (F.A.S.T.) y microscopio de fluorescencia LED en comparación con la microscopía de campo brillante y Giemsa validada por la reacción en cadena de la polimerasa.

Sean M. Parsel, Steven A. Gustafson, Edward Friedlander, Alexander A. Shnyra, Aderosoye J. Adegbulu, Ying Liu, Nicole M. Parrish, Syed A. Jamal, Eve Lofthus, Leo Ayuk, Charles Awasom, Carolyn J. Henry, Carole P. McArthur

Resumen

Antecedentes: El paludismo es un problema grave de salud a nivel mundial y la carga sostenida que representa se debe, en parte, a las dificultades para diagnosticar la enfermedad. La Organización Mundial de la Salud recomienda realizar pruebas de confirmación usando las técnicas de diagnóstico microscópico o las pruebas de diagnóstico rápido (PDR) para todos los casos en que sospeche la existencia de paludismo. En las regiones en las que las especies de *Plasmodium* son autóctonas, hay etiologías de síndromes febriles múltiples que conducen a un diagnóstico erróneo, especialmente en los niños y en las poblaciones en las que prevalece el VIH. Para determinar la frecuencia de la infección palúdica en pacientes febriles, durante 8 meses, en el Regional Hospital de Bamenda, Camerún, evaluamos la eficacia clínica de la Tecnología de Tinción y Fluorescencia (F.A.S.T.) y el sistema con microscopio (FM) ParaLens Advance^{TM}, y la comparamos con el microscopio de campo brillante y la tinción de Giemsa (*Giemsa stain, GS*) convencionales.

Métodos: Se evaluaron muestras de sangre periférica de 522 pacientes con un diagnóstico clínico de "paludismo presunto" usando los métodos GS y FM. El criterio de referencia para comparar los dos métodos fue una prueba de reacción de cadena de la polimerasa (PCR, por sus siglas en inglés) anidada. Se determinó la positividad, la sensibilidad, la especificidad, el valor predictivo positivo (VPP) y el valor predictivo negativo (VPN).

Resultados: en el análisis final se incluyeron 499 muestras. De ellas, 30 fueron positivas mediante la PCR (6,01 %) con un VPP medio de 19,62 % y un 27,99 % para GS y FM, respectivamente.
El VPN fue del 95,01 % y del 95,28 % para GS y FM, respectivamente.
La sensibilidad fue del 26,67 % en ambos grupos, y la especificidad fue del 92,78 % y de un 96,21 % para GS y FM, respectivamente. Los técnicos observaron un nivel aumentado de discrepancia en el diagnóstico según el nivel de habilidad al usar GS, lo que no se vio con FM.

Conclusiones: La frecuencia de las infecciones palúdicas confirmadas mediante la PDR entre los pacientes con fiebre y otros síntomas de paludismo fue mucho menor que la prevista basándose en la sospecha clínica de los médicos. La correlación entre la capacidad de los técnicos y la exactitud del diagnóstico de paludismo usando GS se observó que fue menos pronunciada usando FM.

Además, el método FM aumentó la especificidad y mejoró el VPP, lo que sugiere que este abordaje de costo relativamente bajo podría ser útil en entornos con recursos limitados. Como anécdota, los médicos se mostraban reacios a no tratar a todos los pacientes según los síntomas que presentaban antes de conocer los resultados y a pesar de un diagnóstico microscópico negativo, lo que resalta la necesidad de una formación mejor para los médicos a fin de evitar el sobretratamiento. Se está planificando un estudio más amplio en un área con una alta prevalencia conocida para comparar los dos métodos de microscopía con las RDT disponibles.

Translated from English version into Spanish by María Luisa Belén, through

TRANSLATORS
WITHOUT BORDERS
